# Ambulatory Pessary Trial Unmasks Occult Stress Urinary Incontinence

**DOI:** 10.1155/2012/392027

**Published:** 2011-09-22

**Authors:** Bilal Chughtai, Sara Spettel, Jonathan Kurman, Elise De

**Affiliations:** Continence Center, Urological Institute of Northeast New York and Division of Urology, Albany Medical College, Albany, NY 12208, USA

## Abstract

*Objective*. We evaluated the use of a one-week ambulatory pessary trial in predicting patients' postoperative outcomes for occult stress incontinence. *Methods*. Patients with anterior vaginal wall prolapse were offered a pessary trial to predict response to reconstruction. We performed a retrospective review of 4 years of cases. All patients underwent a detailed evaluation including videourodynamics with and without pessary reduction. *Results*. Twenty-six patients completed the 1-week pessary trial. Ten (38%) women showing no evidence of stress urinary incontinence (SUI) underwent surgical repair of prolapse without anti-incontinence procedure. None of these patients had SUI postoperatively. Sixteen women (61%) had occult stress urinary incontinence on evaluation and underwent concurrent sling procedure. Three (19%) of these patients were identified by the pessary trial alone. Twenty-five of the 26 patients were without clinical stress incontinence at a mean follow up of 12 months (range 4–37 months). The pessary trial correctly predicted persistent urgency in six patients and persistent frequency in five. No patients with SUI or persistent voiding difficult were missed in a pessary trial. *Conclusion*. An ambulatory pessary trial is an effective, easy, and inexpensive method to approximate anatomic results achieved by surgery under real-life conditions. In our series, 20% of patients with occult SUI were identified by pessary trial alone.

## 1. Introduction

Each year, approximately 200,000 women undergo surgical treatment for pelvic organ prolapse (POP) in the United States [1]. Of these women, approximately 21% included urinary incontinence procedures, for an annual cost of greater than $1 billion for surgical repair of prolapse [2]. The demand for POP repair is expected to increase as the U.S. population ages and life expectancies increase. 

The central question in the preoperative evaluation of a patient with POP is to estimate functional outcome once the anatomy is corrected. It is well documented that stress-continent women with advanced POP may develop stress urinary incontinence (SUI) following prolapse reduction [3, 4]. It is thought that correction of the anatomy will unkink or decrease resistance to the urethra, thereby unmasking intrinsic sphincteric deficiency. Regardless of objective outcome of prolapse repair, patient satisfaction with surgery is highly correlated with patient expectations preoperatively [5]. 

There are no clear guidelines regarding concurrent anti-incontinence procedures during surgical prolapse repair. Some surgeons place a sling or perform a retropubic suspension “prophylactically” at the time of all significant prolapse surgery [6, 7]. Others feel this exposes the patient to additional morbidity without proven benefit [8, 9]. Alternatively, it is extremely discouraging for both the patient and surgeon when a patient develops new-onset SUI after having just undergone a major vaginal reconstruction. An additional anti-incontinence procedure necessitates a repeat trip to the operating room, repeat anesthesia, additional recovery period, and surgery in a previously operated field.

Ideally one could predict the need for anti-incontinence surgery at the time of prolapse reduction in women who do not have stress incontinence preoperatively, as well as predict improvement in other urinary symptoms. 

Our study, while not attempting to definitively answer the complex issue of concomitant anti-incontinent surgery during prolapse repair, aims to describe our experience with an ambulatory pessary trial in addition to preoperative urodynamic testing (UDS). Our primary objective is to determine if an ambulatory pessary trial can identify women with occult stress urinary incontinence before prolapse repair. 

There is limited literature examining outcomes with an ambulatory pessary trial. This exercise approximates the anatomic result achieved by surgery under real-life conditions. The trial allows for identification of occult stress incontinence during activities of daily life in the patient's home environment and allows appropriate expectations regarding functional urinary symptoms after surgery. We present the study not as the definitive answer to this controversial surgical question but rather as a tool in the preoperative assessment which we have found clinically useful in our practice.

## 2. Methods

Following Institutional Review Board approval, a retrospective chart review of patients in the Albany Medical Center Division of Urology Clinic and Urodynamic Database was conducted. The electronic medical records of those patients who underwent a pessary trial with a subsequent sling/suspension procedure between June 2005 and February 2009 were identified. All data was tabulated in a deidentified format. Data was collected on patient demographics and the results of urodynamic studies. VUDS data included intra-abdominal, detrusor, and intravesical pressures as well as fill rate at both baseline and maximal capacity. Criteria for inclusion consisted of Baden-Walker grade 2 or higher anterior vaginal wall prolapse and an unresolved diagnostic concern (occult stress incontinence, incomplete emptying, or urge incontinence) and the capacity to retain a pessary. 

All patients underwent a detailed history, physical including pelvic exam, including meticulous multichannel VUDS with and without reduction preoperatively. Urodynamic testing was conducted with a Triton Multichannel Urodynamics Monitor (Laborie, Inc., Burlington, Vt, USA) according to the specifications of the ICS [10]. The urodynamic assessment was performed with the patient sitting with SUI evaluated by having the patient cough and perform a Valsalva maneuver at 200 cc and capacity. Vesical leak point pressure was defined as the minimum amount of pressure necessary to produce visible or fluoroscopic urine leakage. Pressure flow studies were conducted as patients voided after reaching functional capacity. Urodynamic stress incontinence was defined as observable urine leakage during valsalva without associated detrusor overactivity. 

A split speculum technique with the patient in the lithotomy position was utilized to evaluate the extent of the prolapse for classification according to the Baden and Walker criteria [11]. Urethral hypermobility was defined as a change in the urethral angle between rest and straining of 30 degrees. 

Patients who met inclusion criteria were offered a home pessary trial to predict response to reconstruction. The pessary, either Gehrung, donut, or ring, was fitted so it would be large enough to remain in place during periods of increased intraabdominal pressure but loose enough to avoid urethral obstruction. Patients attempted an ambulatory pessary trial for a minimum of one week prior to surgical intervention. Patients were instructed to note their symptoms with the pessaries in place for the duration of the week, while performing all of their usual activities. Patients were followed with respect to clinical symptoms.

Surgical repair of the prolapse was performed by a single surgeon; an additional anti-incontinence procedure, TOT mid-urethral sling, was performed if SUI was identified preoperatively by the ambulatory pessary trial. All patients received a postoperative examination, systematic interview of voiding symptoms and measurement of postvoid residual (PVR). We do not routinely use postoperative urodynamic evaluation. Outcome was based on patient report of leakage postoperatively.

## 3. Results

Between June 2005 and February 2009, 41 patients accepted the home pessary trial. Of these women, 26 were able to retain their pessary for at least one week; subsequent analysis is based on this subset. The mean age of the study subjects was 65 (range 44 to 80). Twenty-four of the women presented with a cystocele, while 10 had a rectocele. The median cystocele grade was Baden-Walker 2 (range 2–4), while the median rectocele grade was 1.8. The median vault grade was 2 (range 2–4), while the mean degree of urethral hypermobility was 39 (range 0–45). Approximately 62% (16) of the patients had a grade 2 cystocele, while 27% (7) had a grade 3. Only one patient (4%) had a grade 4 cystocele. 

Ten (38%) women showed no evidence of SUI by pessary trial, clinical report, VUDS, or physical exam and underwent surgical repair of their prolapse without an accompanying anti-incontinence procedure. None of these women had stress urinary incontinence postoperatively. No intraoperative complications occurred during the operations, listed in Table 2.

Sixteen (61%) women were found to have occult stress urinary incontinence by pessary trial, clinical report, VUDS, or physical exam and underwent a concomitant vaginal sling procedure (Table 3). Three (19%) of these sixteen were identified by the pessary trial alone; their SUI was not detected with VUDS (Table 1). 

The ambulatory pessary trial correctly predicted persistent urgency and persistent frequency in 5 and 6 patients, respectively. Overall, significant decreases in clinical SUI and urge urinary incontinence (UUI) were seen postoperatively (Table 1). 

Twenty-five of the 26 patients who qualified for the study were without clinical stress incontinence after surgery at a mean followup of 12 months (range 4–37 months). 

There were no patients with occult stress urinary incontinence or persistent voiding difficulty whose symptoms were missed in a successful pessary trial.

## 4. Discussion

Occult SUI is a relatively common occurrence in women with severe pelvic organ prolapse and is critical to identify when planning a surgical repair. Our study confirms this finding, as over 60% of the patients had evidence of SUI, 20% of which was occult and identified by pessary trial only. 

The one failure with postoperative SUI occurred in the sling group; although initially dry postoperatively, marked noncompliance with postoperative activity restrictions likely resulted in sling migration. She was later rendered dry by transurethral bulking agent. 

Several studies assert that preoperative VUDS with prolapsed reduction is useful for estimating the risk of developing postoperative incontinence [4, 9, 12]. Surgeons adhering to this philosophy will perform an additional anti-incontinence procedure only in those who show urodynamic stress incontinence. Liang et al. reported that none of their 30 patients who were stress-continent during pessary-reduced urodynamic trial developed SUI postoperatively. They concluded that concomitant anti-incontinence surgery is not necessary in this group [12]. Klutke and Ramos found the same results and arrived at a similar conclusion in a retrospective review of 70 patients [13]. Alternatively, patients who do develop incontinence during prolapse-reduced urodynamics are prone to develop stress incontinence postoperatively if an anti-incontinence procedure is not performed concomitantly [14].

However, the study by Visco et al. showed preoperative use of VUDS is not 100 percent sensitive in identifying occult SUI and its sensitivity is also influenced by which reduction method is used [4]. The pessary was found to be the least sensitive method in detection of masked stress incontinence during urodynamic testing, while the speculum was most sensitive [4]. Although commonly used, the vaginal gauze pack was shown in a single institution series to not be particularly successful at unmasking SUI [14]. Although not particularly sensitive for stress incontinence, preoperative pessary testing has been shown to be highly predictive of postoperative voiding function [8]. Reduction by pessary, however, is relatively easy to perform, convenient, and comfortable for most women [9].

In our retrospective review, we confirmed our hypothesis that an ambulatory pessary trial increases the detection rate of SUI. Multichannel VUDS has been shown to detect most cases of occult SUI; however, a certain percentage will be missed, (20% in our study.) We hypothesize that this failure may be due to the nonphysiologic nature of the UDS testing environment and unmasked by both the length of time and different conditions an ambulatory trial allows. In an effort to reduce the hardships of missing occult SUI, we suggest a home pessary trial for women with severe pelvic organ prolapse with no evidence of SUI during VUDS. By having the anti-incontinence procedure performed concurrently with the prolapse repair, women can avoid the risk and significant dissatisfaction associated with an additional operation. 

In addition to detecting occult SUI that would most likely otherwise be missed, a home pessary trial confers a number of other benefits. It can help predict persistent incomplete emptying, as well as persistent UUI, thereby providing women with appropriate postoperative expectations. In our trial, none of the patients with either of these two conditions were missed during an ambulatory pessary trial. Rather than trying to address refractory UUI postoperatively, a low-cost, low-morbidity pessary trial can provide the clinician and patient with essential prognostic information. 

Our study does contain several limitations, namely, the small sample size, retrospective data collection, reliance on systemic interview rather than standardized pad weight, and limited followup. Also as noted in the results, a significant number of patients were unable to retain for the one-week pessary trial, which does limit its use in preoperative evaluation in patients with perineal relation. Previous studies addressing pessary use report a success rate of 50–71%, depending on patient type and length of trial [15–17]. Although the one-week trial utilized in this study appeared to suffice, the ideal length of a home pessary trial has not been determined. The differences between pessary types could also be a cofounding variable although previous studies found no difference on VUDS between a Smith-Hodge pessary and a ring pessary [18]. 

Given that the prevalence of pelvic organ prolapse and demand for surgical repair is likely to increase; further research is needed to address the preoperative evaluation with larger study populations, prospective data and extended followup. The need for continued research to define appropriate preoperative evaluation and evaluate results of surgical repair for this common condition is obvious.

## 5. Conclusions

A properly fitted pessary will approximate the anatomic result achieved by surgery during activities of daily life. This reversible trial aids in the decision to perform anti-incontinence procedures and in setting appropriate postoperative expectations regarding urgency and emptying ability. In our series, 20% of patients in our stress incontinent group were identified by pessary trial alone. The pessary is a valuable diagnostic tool, and we suggest a home pessary trial for women with pelvic organ prolapse with no evidence of SUI during VUDS.

## Figures and Tables

**Table 1:** Patients demonstrating SUI, UUI, urgency, frequency, and nocturia preoperatively during UDS, pessary trial, and postoperatively.

	Preoperative clinically	UDS	Ambulatory pessary trial	Postoperative clinically
	% (N)	% (N)	% (N)	% (N)
SUI	50 (13)	23 (6)	61 (16)	4 (1)
UUI	85 (22)	38 (10)	15 (4)	23 (6)
Urgency	85 (22)		23 (6)	23 (6)
Frequency	81 (21)		19 (5)	19 (5)
Nocturia	69 (18)			4 (1)

**Table 2:** Procedures performed for correction of prolapse and urinary incontinence.

Procedures	N
Colpocleisis—Le Fort	1
Anterior colporrhaphy	15
Combined AP colporrhaphy	5
Colpopexy—abdominal approach	2
Colpopexy—intravaginal approach	2
Sling repair	14
Laparoscopic colpopexy	1

**Table 3:** Systemic clinic interview.

(1) How often do you usually urinate during the day?	
(2) How many times do you usually urinate during the day?	
(3) How often do you usually urinate during the night?	
(4) How many times do you usually urinate at night? (from time you go to bed until time you wake up for the day)	
(5) What is the reason that you usually urinate?	
(6) Once you get the urge or desire to urinate, how long can you usually postpone it comfortably?	
(7) How often do you get a sudden urge or desire to urinate that makes you want to stop what you are doing and rush to the bathroom?	
(8) How often do you get a sudden urge or desire to urinate that makes you want to stop what you are doing and rush to the bathroom but you do not get there in time? (leak urine or wet pads)	
(9) How often do you experience urine leakage when you sneeze or cough?	
(10) How often do you experience urine leakage when you lift and bend?	
(11) How often do you experience urine leakage when you change positions?	
(12) How often do you experience urine leakage related to physical activity?	
(13) How often do you wet yourself, your pads or your clothes without any awareness of how or when it happened?	
(14) In your opinion how good is your bladder control?	
(15) How often do you have a sensation of not emptying your bladder completely?	
(16) How often do you stop and start during urination?	
(17) How often do you have a weak urinary stream?	
(18) How often do you push or strain to begin urination?	
(19) How bothered are you by your bladder symptoms?	

## References

[1] Boyles SH, Weber AM, Meyn L (2003). Procedures for pelvic organ prolapse in the United States, 1979–1997. *American Journal of Obstetrics and Gynecology*.

[2] Subak LL, Waetjen LE, Van Den Eeden S, Thom DH, Vittinghoff E, Brown JS (2001). Cost of pelvic organ prolapse surgery in the United States. *Obstetrics and Gynecology*.

[3] Richardson DA, Bent AE, Ostergard DR (1983). The effect of uterovaginal prolapse on urethrovesical pressure dynamics. *American Journal of Obstetrics and Gynecology*.

[4] Visco AG, Brubaker L, Nygaard I (2008). The role of preoperative urodynamic testing in stress-continent women undergoing sacrocolpopexy: the Colpopexy and Urinary Reduction Efforts (CARE) randomized surgical trial. *International Urogynecology Journal and Pelvic Floor Dysfunction*.

[5] Elkadry EA (2003). Patient-selected goals: a new perspective on surgical outcome. *American Journal of Obstetrics and Gynecology*.

[6] Raz S, Little NA, Juma S, Sussman EM (1991). Repair of severe anterior vaginal wall prolapse (grade IV cystourethrocele). *Journal of Urology*.

[7] Cross CA, Cespedes RD, McGuire EJ, Zimmern PE (1997). Treatment results using pubovaginal slings in patients with large cystoceles and stress incontinence. *Journal of Urology*.

[8] Lazarou G, Scotti RJ, Mikhail MS, Zhou HS, Powers K (2004). Pessary reduction and postoperative cure of retention in women with anterior vaginal wall prolapse. *International Urogynecology Journal and Pelvic Floor Dysfunction*.

[9] Chaikin DC, Groutz A, Blaivas JG (2000). Predicting the need for anti-incontinence surgery in continent women undergoing repair of severe urogenital prolapse. *Journal of Urology*.

[10] Haylen BT, De Ridder D, Freeman RM (2010). An international urogynecological association (IUGA)/international continence society (ICS) joint report on the terminology for female pelvic floor dysfunction. *Neurourology and Urodynamics*.

[11] Baden WF, Walker TA (1972). Physical diagnosis in the evaluation of vaginal relaxation. *Clinical Obstetrics and Gynecology*.

[12] Liang CC, Chang YL, Chang SD, Lo TS, Soong YK (2004). Pessary test to predict postoperative urinary incontinence in women undergoing hysterectomy for prolapse. *Obstetrics and Gynecology*.

[13] Klutke JJ, Ramos S (2000). Urodynamic outcome after surgery for severe prolapse and potential stress incontinence. *American Journal of Obstetrics and Gynecology*.

[14] Gilleran JP, Lemack GE, Zimmern PE (2006). Reduction of moderate-to-large cystocele during urodynamic evaluation using a vaginal gauze pack: 8-Year experience. *BJU International*.

[15] Clemons JL, Aguilar VC, Tillinghast TA, Jackson ND, Myers DL (2004). Risk factors associated with an unsuccessful pessary fitting trial in women with pelvic organ prolapse. *American Journal of Obstetrics and Gynecology*.

[16] Donnelly MJ, Powell-Morgan S, Olsen AL, Nygaard IE (2004). Vaginal pessaries for the management of stress and mixed urinary incontinence. *International Urogynecology Journal and Pelvic Floor Dysfunction*.

[17] Hanson LAM, Schulz JA, Flood CG, Cooley B, Tam F (2006). Vaginal pessaries in managing women with pelvic organ prolapse and urinary incontinence: Patient characteristics and factors contributing to success. *International Urogynecology Journal and Pelvic Floor Dysfunction*.

[18] Mattox TF, Bhatia NN (1994). Urodynamic effects of reducing devices in women with genital prolapse. *International Urogynecology Journal*.

